# Supplementary material for: Comparison between Fourth-Generation FloTrac/Vigileo System and Continuous Thermodilution Technique for Cardiac Output Estimation after Time Adjustment during Off-Pump Coronary Artery Bypass Graft Surgery: A Retrospective Cohort Study
Source: J Clin Med. 2022 Oct 16;11(20):6093. doi: 10.3390/jcm11206093 (PMC9605331; doi:10.3390/jcm11206093)

**Supplementary File S1. The results of the sensitivity analysis**

**1. Results from dataset with *six-minute time adjustment***

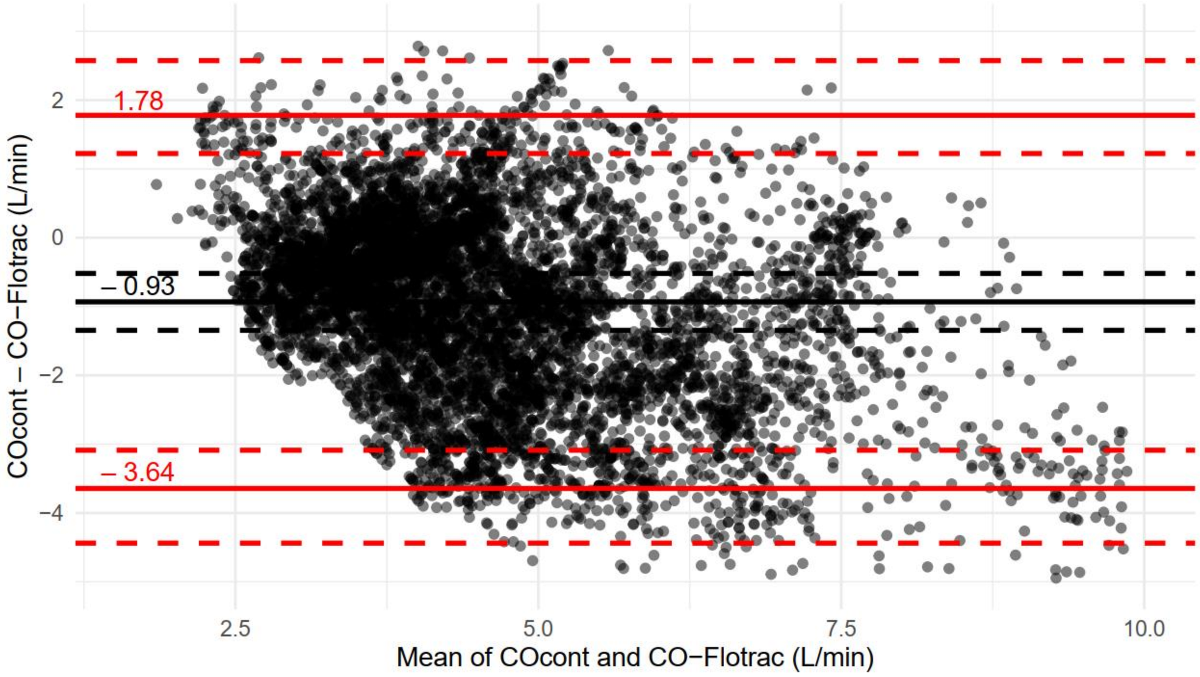


The result of Bland-Altman analysis is shown in the figure above. The mean bias was –0.93 (95% CI, –1.35 to –0.52) L/min and the limits of agreements were –3.64 (95% CI, –4.44 to –3.09) L/min and 1.78 (95% CI, 1.22 to 2.57) L/min. Percentage error was 66.4% (95% CI, 52.8 to 85.8%).

Four-quadrant plots using three- to 20-minutes interval change of $CO_{cont}$ and CO-FloTrac are shown in the figure below. Depending on the time scale and the size of the exclusion zone, concordance rates were ranged from 58.5 to 72.8%.

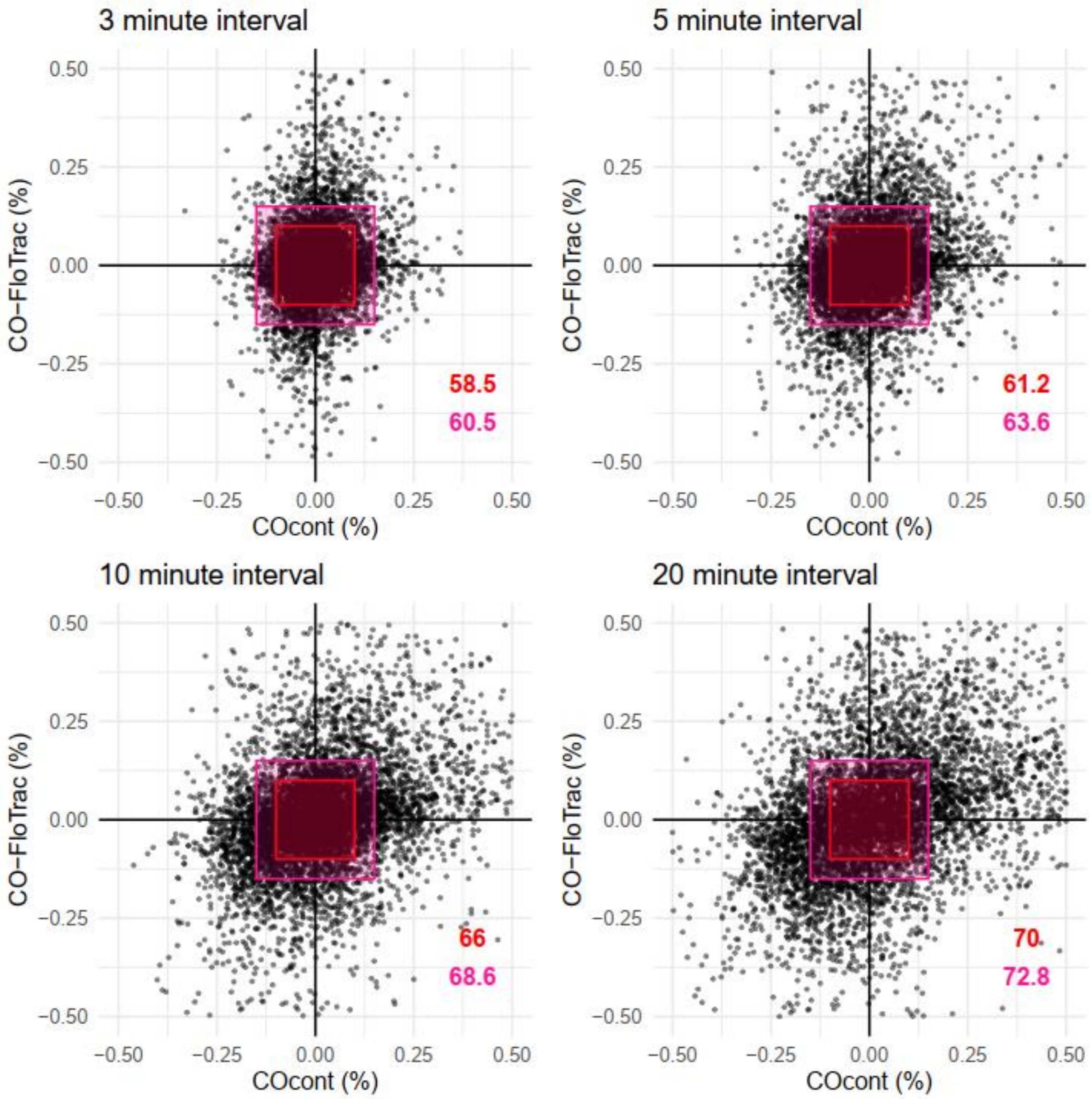

## 2. Results from dataset *without time adjustment*

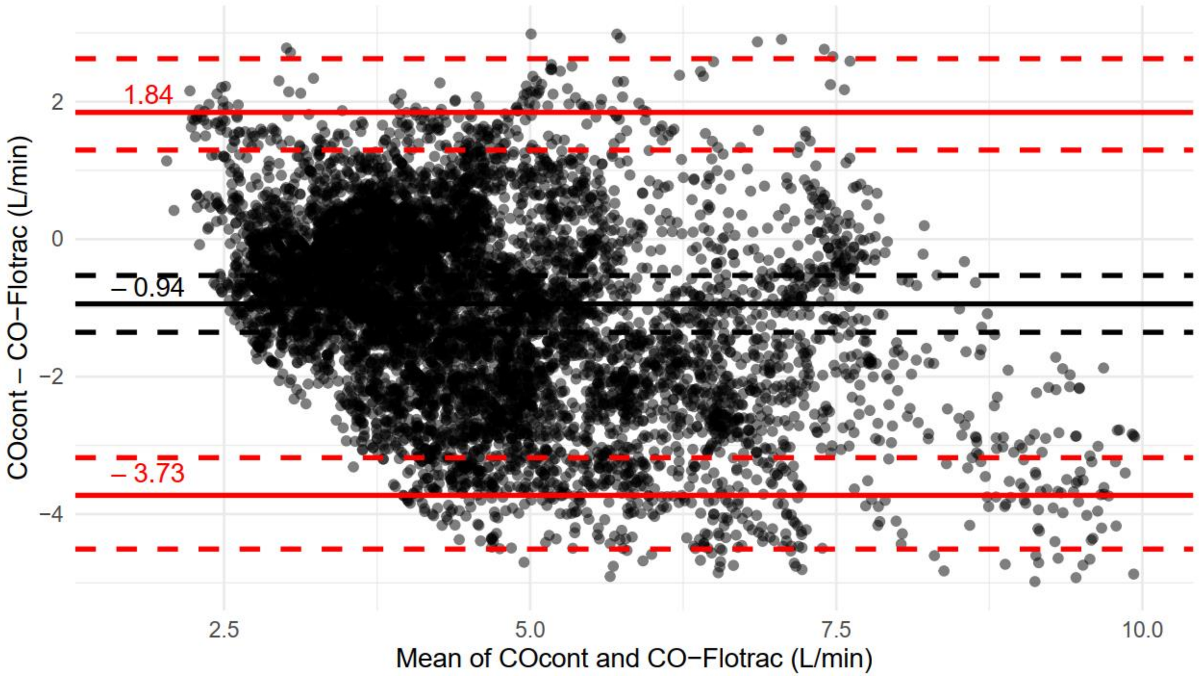


The result of Bland-Altman analysis is shown in the figure above. The mean bias was –0.94 (95% CI, –1.35 to –0.53) L/min and the limits of agreements were –3.73 (95% CI, –4.51 to –3.18) L/min and 1.84 (95% CI, 1.30 to 2.63) L/min. Percentage error was 68.2% (95% CI, 54.8 to 87.3%).

Four-quadrant plots using three- to 20-minutes interval change of $CO_{cont}$ and CO-FloTrac are shown in the figure below. Depending on the time scale and the size of the exclusion zone, concordance rates were ranged from 48.2 to 66.1%.

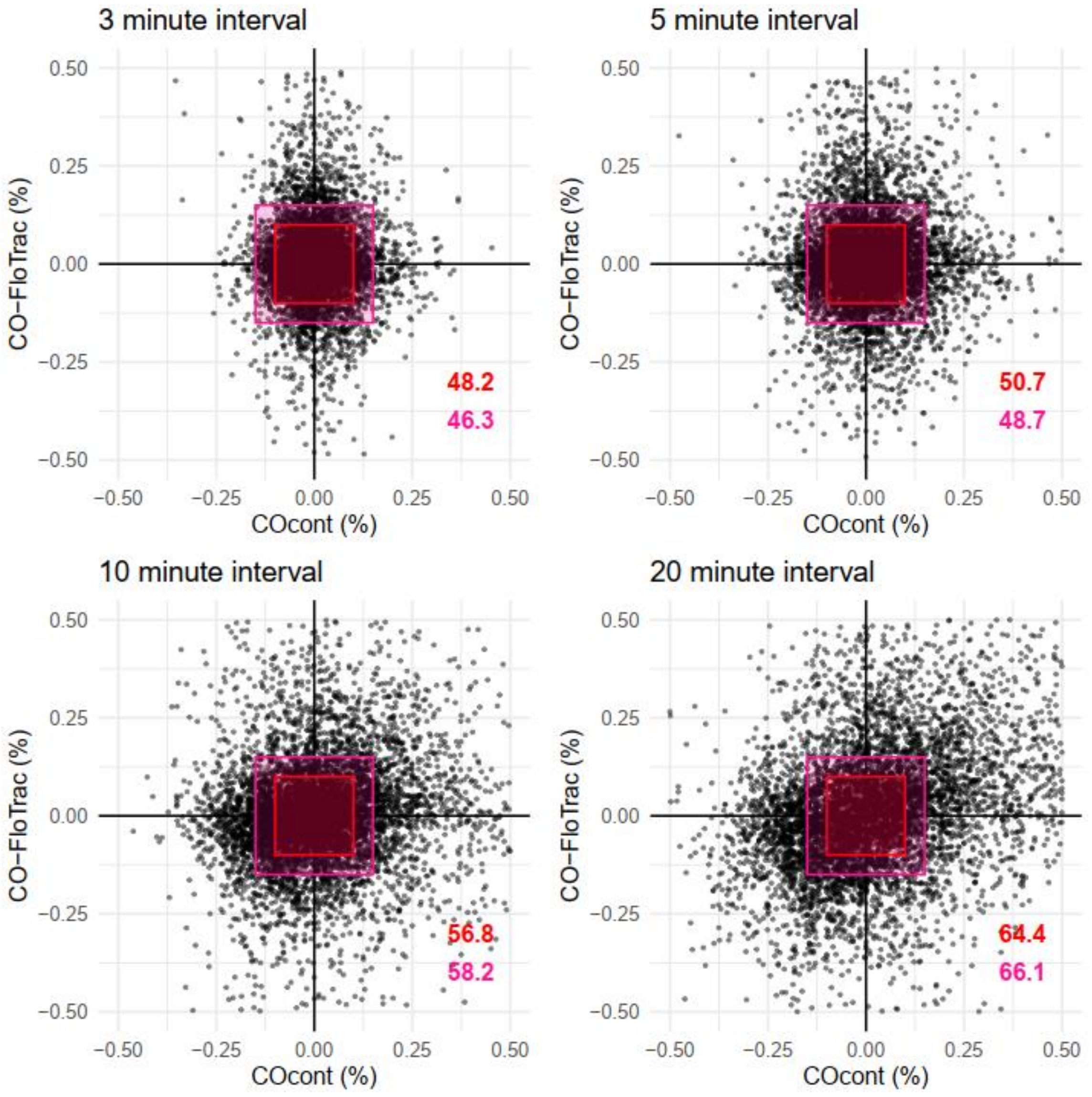

Supplement: Supplementary file 1 [file jcm-11-06093-s001.zip › jcm-1931947-supplementary.pdf]
